# Evaluation and Verification of a microRNA Panel Using Quadratic Discriminant Analysis for the Classification of Human Body Fluids in DNA Extracts

**DOI:** 10.3390/genes14050968

**Published:** 2023-04-25

**Authors:** Ciara Rhodes, Carolyn Lewis, Kelsey Price, Anaya Valentine, Mary-Randall A. Creighton, Edward Boone, Sarah Seashols-Williams

**Affiliations:** 1 Department of Forensic Science, Virginia Commonwealth University, P.O. Box 843079, 1015 Floyd Ave., Richmond, VA 23284-3079, USA; 2 Integrative Life Sciences Program, Virginia Commonwealth University, P.O. Box 842030, 1000 West Cary St., Richmond, VA 23284-2030, USA; 3 Center for Biological Data Science, Virginia Commonwealth University, P.O. Box 842030, 1015 Floyd Ave., Richmond, VA 23284-2030, USA; 4 Department of Statistical Sciences and Operations Research, Virginia Commonwealth University, P.O. Box 843083, 1015 Floyd Ave., Richmond, VA 23284-3083, USA

**Keywords:** forensic science, body fluid identification, microRNA, quadratic discriminant analysis (QDA)

## Abstract

There is significant interest in the use of miRNA analysis for forensic body fluid identification. Demonstrated co-extraction and detection in DNA extracts could make the use of miRNAs a more streamlined molecular body fluid identification method than other RNA-based methods. We previously reported a reverse transcription-quantitative PCR (RT-qPCR) panel of eight miRNAs that classified venous and menstrual blood, feces, urine, saliva, semen, and vaginal secretions using a quadratic discriminant analysis (QDA) model with 93% accuracy in RNA extracts. Herein, miRNA expression in DNA extracts from 50 donors of each body fluid were tested using the model. Initially, a classification rate of 87% was obtained, which increased to 92% when three additional miRNAs were added. Body fluid identification was found to be reliable across population samples of mixed ages, ethnicities, and sex, with 72–98% of the unknown samples classifying correctly. The model was then tested against compromised samples and over biological cycles, where classification accuracy varied, depending on the body fluid. In conclusion, we demonstrated the ability to classify body fluids using miRNA expression from DNA extracts, eliminating the need for RNA extraction, greatly reducing evidentiary sample consumption and processing time in forensic laboratories, but acknowledge that compromised semen and saliva samples can fail to classify properly, and mixed sample classification remains untested and may have limitations.

## 1. Introduction

DNA evidence is a valuable forensic tool that can place persons involved in a crime at the scene or tie them to evidence. However, body fluid identification is still important for corroborating testimony and lending additional information about the specific events of an alleged crime, especially in violent crimes, such as homicide or sexual assault. These limitations indicate a need for more accurate body fluid identification assays which can accurately identify all forensically relevant body fluids and do not contribute to unnecessary sample consumption.

MicroRNAs are small, non-coding RNAs that range from 21 to 24 nucleotides long, and because of this, are less susceptible to degradation as compared to longer mRNAs [1,2,3,4,5,6]. Many miRNAs are differentially expressed in body fluids, and the expression levels of these diagnostic markers can be evaluated to identify forensically relevant body fluids [6,7,8,9,10,11,12,13,14,15,16,17,18,19,20]. One challenge of traditional RNA assays is the need for a separate RNA extraction, as it unnecessarily consumes the sample [21]. However, miRNAs can be co-extracted with DNA using several commonly used DNA extraction methods without the need for an extra DNase treatment step, which cuts down on time and sample consumption [22,23,24,25,26].

Seashols-Williams et al. [11] previously reported a reverse transcription-quantitative PCR (RT-qPCR) panel of eight miRNAs in RNA extracts that classified venous and menstrual secretions, feces, urine, saliva, semen, and vaginal secretions through analysis of differential expression. This panel of miRNAs includes a pair of endogenous reference markers that provide normalization of miRNA expression without evaluation of the RNA quality or known input quantity. In a subsequent report, the expression levels of these markers were validated using blood, semen, saliva, urine, vaginal fluid, menstrual secretions, and perspiration. The ability of this panel to identify body fluids was tested using a quadratic discriminate analysis (QDA) model, which was created using the statistical software R version 4.0.2 (R Foundation for Statistical Computing, Vienna, Austria), and consisted of a ten-fold cross validation of the model. This model used the normalized expression levels obtained from experimental samples to predict the presence of body fluids [10]. The model correctly classified 93.3% of samples when tested in blood, menstrual secretions, feces, urine, saliva, semen, and vaginal fluid. Following these results, the expression levels were investigated within a donor over a biological cycle in RNA extracts. The correct classification rates in blood, feces, urine, and vaginal fluid were comparable to that of the population studies; however, the classification rates of saliva, semen, and menstrual secretions were lower.

To create a more easily implemented assay for forensic DNA laboratories, the presence of miRNA retention and expression in DNA extracts was assessed and found to be only slightly lower than in RNA extracts [26]. Based on those results, we were interested in assessing the ability of our body fluid classification model to identify body fluids using miRNAs retained in DNA extracts.

## 2. Materials and Methods

### 2.1. Validation of miRNA Panel for Body Fluid Identification in DNA Extracts

The following sample collection, DNA isolation and RT-qPCR analysis methods apply to all analyses unless stated otherwise.

#### 2.1.1. Sample Collection

Blood, menstrual secretions, feces, urine, saliva, semen, and vaginal secretions were collected between 2016–2020 using informed consent in accordance with the approved Institutional Review Board Human Subjects Research Protocol (HM200009027). Menstrual, fecal, and vaginal samples were collected on sterile cotton swabs by the donors and returned in swab boxes. Blood was deposited onto a sterile cotton swab after sterilizing and pricking the donor’s finger with a Unistik^®^ 3 Normal lancet (Owen Mumford Ltd., Woodstock, UK), and saliva was collected by rolling a sterile cotton swab along the inside of the donor’s cheek. Urine and semen were deposited into sterile collection cups supplied to the donor, which were returned on ice within 24 h before aliquoting each onto sterile cotton swabs (50 µL of semen and 100 µL of urine). All swabs were dried and stored in swab boxes at room temperature until treatment and/or DNA extraction.

#### 2.1.2. DNA Isolation

DNA was isolated from whole swabs using the QIAgen DNA Investigator Kit on the QIAcube (Qiagen, Valencia, CA, USA) and the previously validated manufacturer’s protocol for forensic casework samples. No modifications such as the addition of glycogen were used in an effort to mimic a forensic workflow as closely as possible. Final elution volumes were as follows: 30 μL for saliva, blood, menstrual secretions, semen, and vaginal fluid, 50 μL for feces, and 20 μL for urine. Reagent blanks were included with each batch of DNA extractions, and extracts were stored at −80 °C until further use. DNase treatment was not performed on the sample extracts, as primer evaluation and previous work has demonstrated no significant difference on miRNA detection levels [16,26], indicating that genomic DNA does not interfere with miRNA detection. Reverse Transcription and qPCR controls were analyzed to verify lack of interference in miRNA amplification by genomic DNA.

DNA extractions and subsequent cDNA synthesis were performed in a dedicated workspace for RNA, following strict procedures to help limit the effect of contamination, including physical isolation from post-PCR laboratories, use of personal protective equipment, and use of molecular biology grade reagents and consumables. miRNA isolation efficiency was measured through RT-qPCR analysis, as our previous work has shown that UV spectrophotometry and other methods cannot precisely predict miRNA concentrations in the low concentrations observed in biological fluids [10,11,16,26]. 

#### 2.1.3. Sample Treatment for Compromised Analysis

Blood, semen, saliva, and urine were collected from three different donors. Blood was collected into a Vacutainer^®^ containing EDTA (Beckton, Dickinson & Company, Franklin Lakes, NJ, USA) and inverted for 15 s before 50 μL was deposited onto a sterile cotton swab. Urine, semen, and saliva were collected into a sterile collection cup, and 50 μL (semen, saliva) or 100 μL (urine) were deposited onto sterile cotton swabs. The swabs were dried at room temperature for 24–48 h and then stored at −20 °C until treatment, which was performed within 72 h of drying. 

For heat-compromised samples, swabs were exposed to either 55 °C or 95 °C for 0.5, 1, 2, 4, or 24 h. For the chemically treated samples, 100 μL of either 1:10 (87 nM) or full-strength (870 mM) sodium hypochlorite, dish soap (Dawn Ultra Dishwashing Liquid, Proctor & Gamble, Cincinnati, OH), or glacial acetic acid (pH 2.5, 17.4 M) were deposited onto the prepared swabs and dried for 72 h. UV-treated samples were exposed to 4 h of 302 nm light at room temperature using the UVP high-performance ultra-violet transilluminator (UVP, Upland, CA, USA). This wavelength was chosen as it is in the middle of the ultraviolet range and has been demonstrated to induce the most significant DNA damage [27]. After treatment, all swabs were stored at −20 °C until DNA isolation. 

Dried blood, semen, saliva, and urine were also tested in an environmental chamber for simulated outdoor conditions. Samples were deposited onto a cotton swatch from Layne et al. [16] using a single donor for each body fluid. The samples were exposed to treatment in a Q-sun Ce-3 Environmental Chamber (Q-Lab Corporation, Westlake, OH, USA) at the Federal Bureau of Investigation Research Laboratory. The Environmental chamber controlled for temperature, humidity, and a 24 h light/dark cycle to imitate a summer day in Virginia (Supplementary Table S1). The samples were removed from the chamber at 48 h intervals up to 14 days and stored at −80 °C until punches were taken. Using a biopsy punch, 4 mm punches were taken from the remaining cotton swatches and stored at −80 °C until DNA isolation (5 years).

#### 2.1.4. RT-qPCR

Reverse transcription was performed on the Proflex PCR System (Thermo Fisher Scientific, Waltham, MA, USA) using the qScript™ microRNA Quantification System (Quanta Biosciences, Gaithersburg, MD, USA) following the previously reported protocol [16,26]. qPCR primers for all miRNA target sequences were purchased from IDT (Integrated DNA Technologies, Coralville, IA, USA) (Supplementary Table S2), and qPCR was performed according to the protocol in quarter volume reactions: 6.25 µL of 2X PerfeCTa^®^ SYBR Green SuperMix, 0.25 µL of PerfeCTa^®^ Universal Primer (UP: 5′-ATGGCGGTAAGTCCAGATACG-3′)(Quanta Biosciences) and IDT MicroRNA Primer Assay (2.5 µM), 3.75 µL of nuclease-free water, and 2 µL of cDNA reaction for a total reaction volume of 12.5 µL. Thermal cycling parameters on the QuantStudio™ 6 Flex Real-Time PCR Instrument (Thermo Fisher Scientific) were set at: 95 °C for 2 min, 40 cycles of 95 °C for 5 s, 60 °C for 15 s, and 70 °C for 34 s. Raw data were analyzed at a threshold of 0.015 within QuantStudio™ Real-Time PCR software v1.3 (Thermo Fisher Scientific) and exported into Microsoft Excel (Microsoft Corporation, Redmond, WA, USA). Differential expression or delta quantification cycle (ΔCq) values were calculated by subtracting the average Cq of let-7g and let-7i from the Cq value of the target miRNA (ΔCq = Cq(_miRNA target_) − Cq(_avg let-7g & let-7i_)). 

All experiments were performed and analyzed according to Minimum Information for Publication of Quantitative Real-Time PR Experiments (MIQE) guidelines [28]. Each miRNA target was amplified in duplicate technical replicates for each sample with no template controls (NTCs) and negative reverse transcription (RT) controls (non-transcribed RNA extract—to eliminate genomic DNA contamination as a variable) on each plate.

### 2.2. Statistical Analyses

Initial statistical analyses of the raw and ΔCq data were performed in JMP^®^ v14.2.0 (SAS Institute, Cary, NC, USA). Normal distribution and equal variance were confirmed for all sample sets using quantile–quantile plots and Levine’s test, respectively. In multi-group comparisons, a one-way ANOVA test was performed with Tukey’s HSD pairwise comparison. A significance level of α = 0.05 was used to determine which tests are declared statistically significant. Subsequent statistical prediction modeling was performed in R version 4.0.2 (R Foundation for Statistical Computing, Vienna, Austria). For predictive analysis, Quadratic Discriminant Analysis (qda in R) [29] was used. A 10-fold cross validation procedure was performed to ensure that each observation was in both training and validation sets by splitting the data into ten non-overlapping groups, corresponding to 10% of the samples. Each group was in turn used as a test set while the remaining (i.e., 90% of the data) were used to train the model. 

Once sample collection was complete, the population dataset consisted of 355 samples: 51 blood, 53 menstrual secretions, 50 feces, 46 urine, 53 saliva, 52 semen, and 50 vaginal secretions (Supplementary Table S3). For some of the markers and body fluid combinations, there were some samples in which not all miRNAs were tested; thus, across the total dataset, there were 252 fully observed values. To address the issues associated with these missing values, a single imputation using the conditional multivariate mean was employed. This allowed for the imputed value to depend on all the observed values for the subject. Multiple imputation was avoided as the goal of the project is correct classification, and multiple imputation would not allow for easy model validation. To improve the ability of the classifiers, an “Other” category was created by fitting a multivariate normal distribution to the entire dataset and drawing samples outside the 3.5-standard-deviation ellipsoid. Without this other category, any future extreme observations will be classified as the body fluid closest even though it is truly an extrapolation. This Other category allows for only those body fluids with measurements in the range observed to be classified as a body fluid. All measurements outside the observed measurement range will be classified as Other. After development of the QDA model and cross-validation, classification testing was set at 50% confidence for body fluid classification.

### 2.3. Variation within Individuals over Time

To evaluate variation in differential expression within an individual over time or within a biological cycle, three volunteers for each body fluid donated multiple samples according to the time conditions listed in Table 1. Sample collection, DNA extraction, and RT-qPCR analysis were performed as described above.

## 3. Results

### 3.1. Validation of miRNA Panel for Body Fluid Identification

Initial evaluation of miRNA detection for body fluid identification in DNA extracts were modeled on our previous work in RNA extracts [10] by testing the same panel of miRNAs (miRs 200b, 320c, 10b, and 891a relative to the average expression of lets-7g and 7i) in 50 population samples for each biological fluid (blood, semen, vaginal and menstrual secretions, saliva, feces and urine). Development, verification, and 10-fold cross validation of an analogous QDA model for DNA extracts demonstrated 88.0% overall accuracy. Identification of the biological fluids was found to be reliable across population samples of mixed ages, ethnicities, and sex, with 72–98% of the unknown samples classified correctly (Supplementary Table S4). As expected, miRNA detection was slightly different in DNA extracts as compared to the RNA extracts previously validated, and so the predictive model was not as accurate as the identification in RNA extracts. Therefore, we identified additional markers to test and improve accuracy of the panel. 

**Figure 1:**
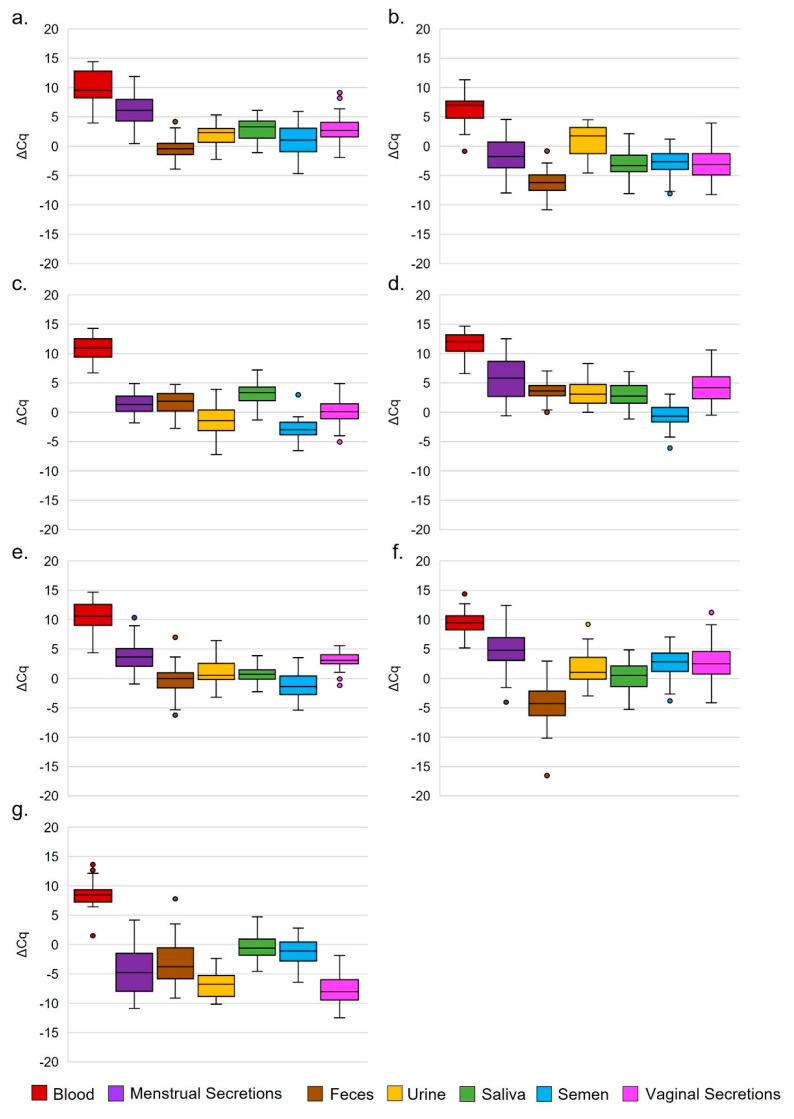
Differential expression patterns (∆Cq values) of miRNAs in an expanded population for the identification of forensically relevant body fluids. (**a**) miR-200b (*n* = 345), (**b**) miR-320c (*n* = 345), (**c**) miR-10b (*n* = 345), (**d**) miR-891a (*n* = 345), (**e**) miR-141 (*n* = 342), (**f**) miR-412 (*n* = 330), and (**g**) miR-205 (*n* = 252). Circles indicate outlying samples.

We identified markers from the literature and our previous work that had the potential to discriminate body fluids in DNA extracts and evaluated them using a tiered population sample approach to conserve samples. We tested several miRNAs identified in the literature with a sample of our population extracts and identified miRs-141, 412, and 205 as being possibly discriminatory and useful to add to the panel. We found that all three miRNAs could assist in improving discrimination, particularly in feces, menstrual and vaginal secretions, saliva (Figure 1) and urine, for an overall prediction accuracy of 92.1% in a 10x-cross-fold validation of the quantitative discriminant analysis (QDA) method (Table 2, Supplementary File S1). We have made this prediction model available for public use (https://vcu-frsc-sswlab.shinyapps.io/QDA-Prediction-Analysis-wDNA/ accessed on 24 April 2023), and the code can be found at https://github.com/VCU-Forensic-Science-Williams-Lab accessed on 24 April 2023 (file name miRNA panel in DNA-7 miRs (1).R). Note that the only disadvantage of including more markers is time and wells in preparation of the qPCR plate, as preparation of the reverse transcription reaction is the same regardless of the number of markers (until the reverse transcription reaction is depleted), and downstream data analysis is also unchanged for the practitioner.

### 3.2. Variation within Individuals over Time

miRNA detection from DNA extracts of each body fluid were measured to observe whether there was a change over the course of biological cycles or time (Table 1). Blood, urine, and vaginal secretions performed similarly in classification rates to the population samples (Table 3), while semen was classified less accurately (77.8% as compared to 90% in population samples). However, menstrual secretions were dramatically decreased in terms of their accurate classification rate, though they were mostly misclassified as vaginal secretions, and none were misclassified as blood. Saliva classification was very poor in these samples—the expression of miR-412 and miR-205 in the saliva samples after eating on the first day were significantly different from the previous donation and the donation directly following it (*p* < 0.05,). The expression of miR-205 was also significantly different in the day 2 wake up donation when compared to the donation before it (*p* < 0.05). The classification rate in these samples was very low due to these differences, which could be caused by differences in metabolism and stimulation of different salivary glands prior to and after eating a meal [30].

### 3.3. Detection of miRNAs in Treated Samples

#### 3.3.1. Heat Treatment

**Figure 2:**
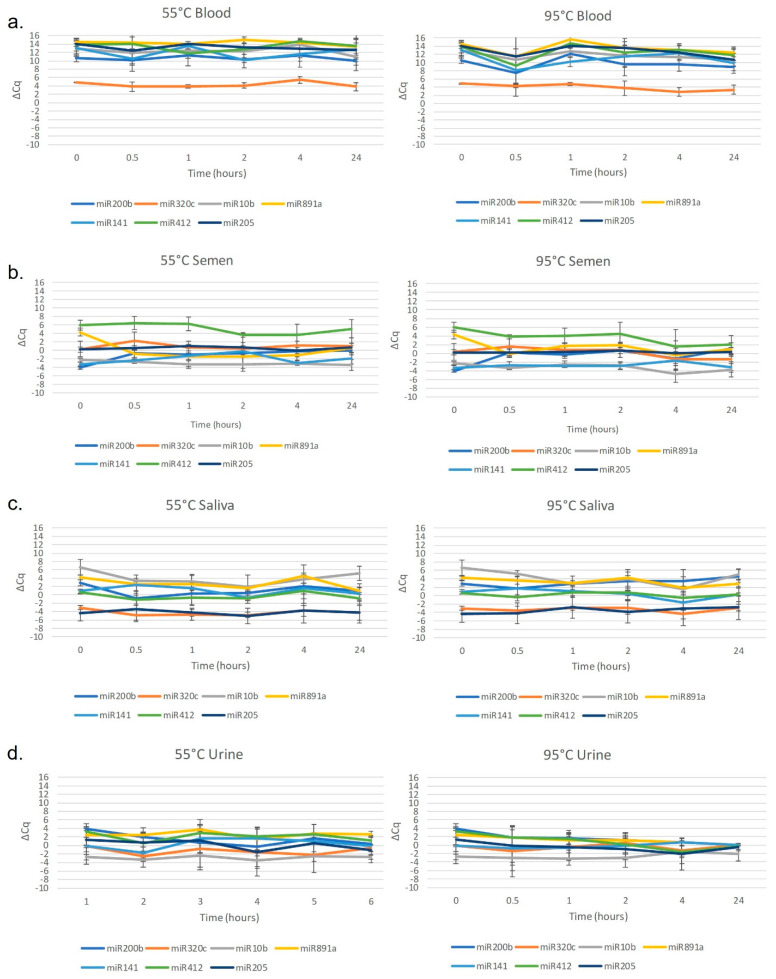
Average dCq values of samples of (**a**). blood, (**b**). semen, (**c**). saliva, or (**d**). urine that were exposed to 55 °C or 95 °C for 0, 0.5, 1, 2, 4, or 24 h in a heat block (*n* = 33). No significant deviations were observed from the control.

The robustness of miRNA marker detection from DNA extracts of blood, semen, saliva, and urine was tested over a period of 24 h at a temperature exposure of either 55 °C or 95 °C. MicroRNA levels in blood proved to be highly resistant to degradation over all heat treatments and showed no significant variation among markers when compared to the untreated controls (Figure 2). These findings are similar to those of Fang et al., which also demonstrated the robustness of miRNA markers in RNA extracts from blood at elevated heat conditions [19], as well as the findings from Layne et al. and Mayes et al. using RNA extracts of blood [9,16]. While urine demonstrated some evidence of degradation, classification rates were similar to the population samples. In contrast, semen and saliva markers were degraded over time after exposure to heat treatment, resulting in a reduced classification rate of 57.6 and 48.5%, respectively, compared to 90 and 84%, respectively, for population classification (Table 4).

#### 3.3.2. Chemical and Ultraviolet Treatment

The effects of chemical or UV treatment on blood, semen, saliva, and urine showed similar degrading patterns, with blood and urine classifying at a rate similar to the population samples, and semen and saliva demonstrating degradation resulting in reduced classification rates (Supplementary Figure S1, Table 4). Semen was most greatly impacted by the application of dish soap, 1:10 bleach dilution, or full-strength bleach. Markers miR200b, miR10b, and miR205 were the most significantly different from the untreated controls after these treatments in semen samples (*p* < 0.05). These differences are reflected in the low correct classification rate of 66.7%. The low classification rate is supported by findings from Mayes et al., which found differing ΔCq values after laundering with a detergent [9]. Saliva exhibited a low classification rate of 33.3% and showed greater sensitivity to UV and glacial acetic acid (GAA) treatment. Saliva also showed significant degradation after full strength bleach treatment in all markers except for miR200b. These findings differ from the results in corresponding RNA extracts in Layne et al., which found that urine was not significantly affected by these treatments [16]. 

#### 3.3.3. Environmental Chamber Stability

Exposure to controlled heat, light/dark cycle, and humidity showed the same pattern, with blood and urine classification unchanged from population samples, indicating the robustness of the markers and classification method. In contrast, neither semen nor saliva samples were correctly classified in any of the treated or untreated samples (Table 4). Since the untreated samples were also incorrectly classified, and showed high raw Cq values, the results for this sample set are inconclusive (Supplementary Figure S2). The age of these samples may have also been a factor, considering that they were stored at −80 °C for five years after treatment, implying that future sample age studies should be evaluated further.

## 4. Discussion

This study investigated the ability to classify body fluids in DNA extracts using a more comprehensive set of miRNA markers than previously evaluated. A total of 355 samples of DNA extracts from blood, semen, vaginal and menstrual secretions, saliva, feces and urine were tested for classification accuracy in the original and subsequently expanded panel of miRNAs, resulting in a QDA model with an overall accuracy of 92%. 

Further evaluation of panel performance in individuals over time and compromised samples demonstrated some limitations of the method in certain biological fluids. Heat treatments had a greater impact on both the detected miRNA quantities in semen and saliva as well as the classification accuracy compared to blood and urine. This trend continued with the chemical and UV treatments; however, the overall decrease in detectability proved to be treatment, body fluid, and marker dependent. Marker expression across a biological cycle appeared to impact correct classification rates dramatically in saliva and menstrual secretions, indicating that the addition of other more consistent markers may be necessary for reliable prediction. As these findings model those results from the same sample types in RNA extracts, our data suggest that detection and prediction in saliva and semen tends to be less robust. Additionally, our previous work in RNA extracts indicated a limitation of the prediction model for handling samples of mixed sources, which of course are often encountered, particularly in sexual assault samples [10]. 

The development of a panel of miRNAs that can predict body fluids with over 90% accuracy from DNA extracts is a significant step forward. By developing a robust method that uses DNA extracts instead of RNA extracts, a significant barrier to implementation is removed—that of additional analyst time, reagent costs, and sample consumption required for a separate RNA isolation method. Much of the historical resistance to a novel body fluid identification method such as mRNA or miRNAs has been due to the additional isolation methods required; therefore, using a DNA extract for body fluid identification combined with analysis methods that utilize existing equipment in a forensic laboratory could lead to rapid, large-scale implementation into the forensic DNA analysis workflow. However, before the assay can be implemented into casework, an evaluation of different analysis methods and prediction modeling in which compromised and mixed samples can be accurately classified is important in order to address real-world sample types. It may also be beneficial to explore adding non-miRNA markers to the panel, such as a combinatorial assay using microbial DNA markers and/or methylation, which may be more accurate than miRNA markers alone given the relative strengths of each marker type. Having now demonstrated that miRNAs are detectable in DNA extracts, this is a possibility, and biomarkers can be combined into a more comprehensive assay [31]. As more markers are added to the panel, targeted high-throughput sequencing may be considered instead of qPCR, since it would allow this assay to be performed more quickly while simultaneously evaluating many markers of different origins. 

## Figures and Tables

**Table 1:** Sampling scheme followed for the miRNA panel analysis addressing variation in differential expression over a biological cycle or time (*n* = 3 unique donors for each sample set).

Body Fluid	Samples Collected
Blood	3–5 donations within a 7-day period
Menstrual Secretions	3–7-day donations
Feces	3 donations within a 7-day period
Urine	6 donations over a 3-day period: upon waking and afternoon
Saliva	3 donations/day for 3 days: upon waking, before a meal, after a meal
Semen	3 donations within a 30-day period (>3 days postcoital activity)
Vaginal Secretions	>21-day donations

**Table 2:** Differential expression of body fluids within the expanded population set were evaluated with the trained QDA model. ΔCq values from the samples were imported into the model. Overall classification percentages are displayed above. (Mens. = menstrual secretions, Vag. = vaginal secretions).

Body Fluid	*n*	Correct Body Fluid Classification	Classification as Another Body Fluid	Classification as Other
Blood	49	97.96%	2.04%	0.00%
Mens.	50	72.00%	28.00%	0.00%
Feces	50	98.00%	2.00%	0.00%
Urine	46	84.80%	15.20%	0.00%
Saliva	50	84.00%	16.00%	0.00%
Semen	50	90.00%	10.00%	0.00%
Vag.	50	72.00%	28.00%	0.00%

**Table 3:** Classification for samples included in the variation within the donors’ sample set. (Mens. = menstrual secretions, Vag. = vaginal secretions).

Body Fluid	*n*	Correct Classification	Classification as Another BF	Classification as Other
Blood	9	1.000	0	0
Mens.	17	0.059	0.941	0
Feces	9	0.778	0.222	0
Saliva	27	0.074	0.926	0
Semen	9	0.778	0.222	0
Urine	18	0.944	0.056	0
Vag.	65	0.769	0.231	0

**Table 4:** Classification rates for treated samples.

Body Fluid	*n*	Correct Classification	Classification as Another BF	Classification as Other
Heat Treated				
Blood	33	0.970	0.030	0.000
Semen	33	0.576	0.424	0.000
Saliva	33	0.485	0.515	0.000
Urine	33	0.848	0.061	0.000
Chem/UV Treated				
Blood	33	1.000	0.000	0.000
Semen	33	0.667	0.333	0.000
Saliva	33	0.333	0.611	0.030
Urine	33	0.778	0.222	0.000
Environmental Chamber				
Blood	8	1.000	0.000	0.000
Semen	8	0.000	0.000	0.000
Saliva	8	0.000	0.000	0.000
Urine	8	0.875	0.125	0.000

## Data Availability

The data presented in this study are available in Supplemental File S1.

## Supplementary Materials

The following supporting information can be downloaded at: https://www.mdpi.com/article/10.3390/genes14050968/s1, Supplementary File S1: population data; Tables S1–S4: Temperatures, primers, demographics and confusion matrix; Figures S1 and S2: treated sample dCq data.
