# Supplementary material for: Evaluation and Verification of a microRNA Panel Using Quadratic Discriminant Analysis for the Classification of Human Body Fluids in DNA Extracts
Source: Genes (Basel). 2023 Apr 25;14(5):968. doi: 10.3390/genes14050968 (PMC10218048; doi:10.3390/genes14050968)
Supplement: Supplementary file 1 [file genes-14-00968-s001.zip › Supp Fig S1.pdf]

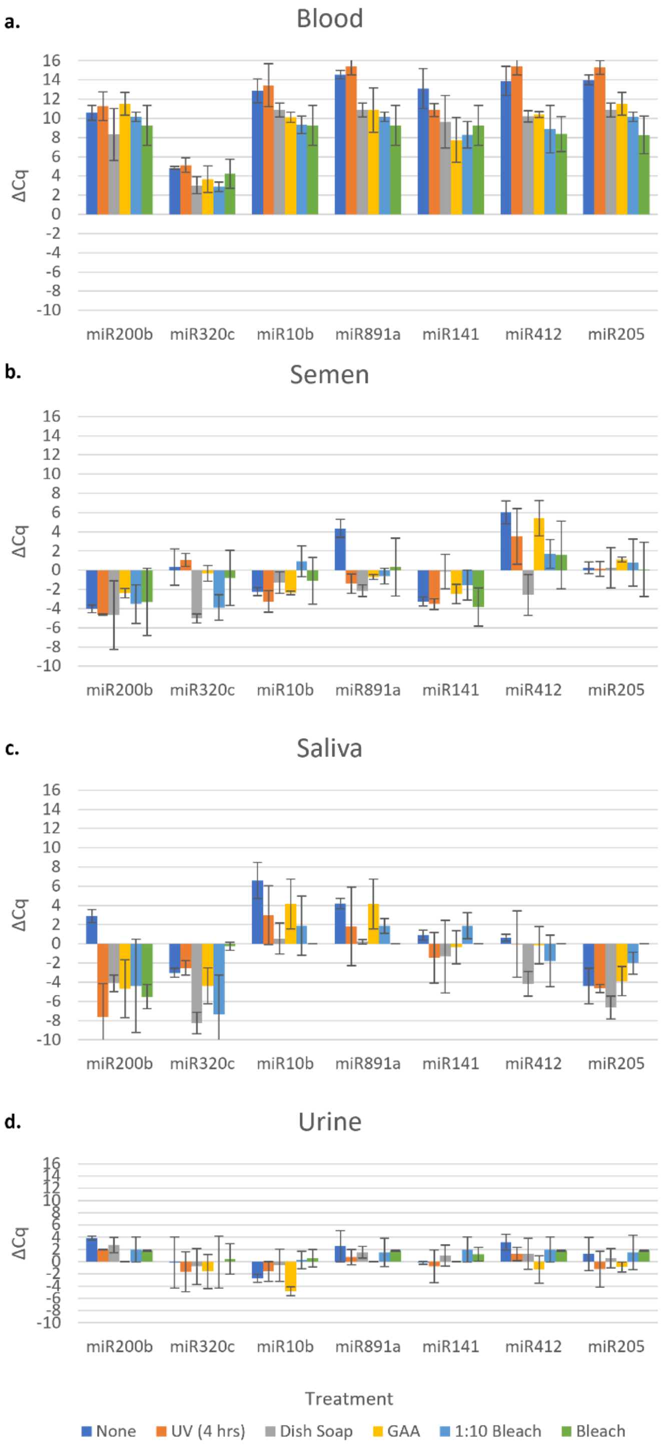

Supp Fig S1. Average dCq values of a. blood, b. semen, c. saliva, or urine, treated with 4 hours of ultraviolet light (UV (4 hrs)), 100 µL of Dawn Dish Soap (dish soap), Glacial Acetic Acid (GAA), 1:10 Bleach dilution or full-strength Bleach (*n*=18 per body fluid).
