# Supplementary material for: Evaluation and Verification of a microRNA Panel Using Quadratic Discriminant Analysis for the Classification of Human Body Fluids in DNA Extracts
Source: Genes (Basel). 2023 Apr 25;14(5):968. doi: 10.3390/genes14050968 (PMC10218048; doi:10.3390/genes14050968)
Supplement: Supplementary file 1 [file genes-14-00968-s001.zip › Supp Fig S2.pdf]

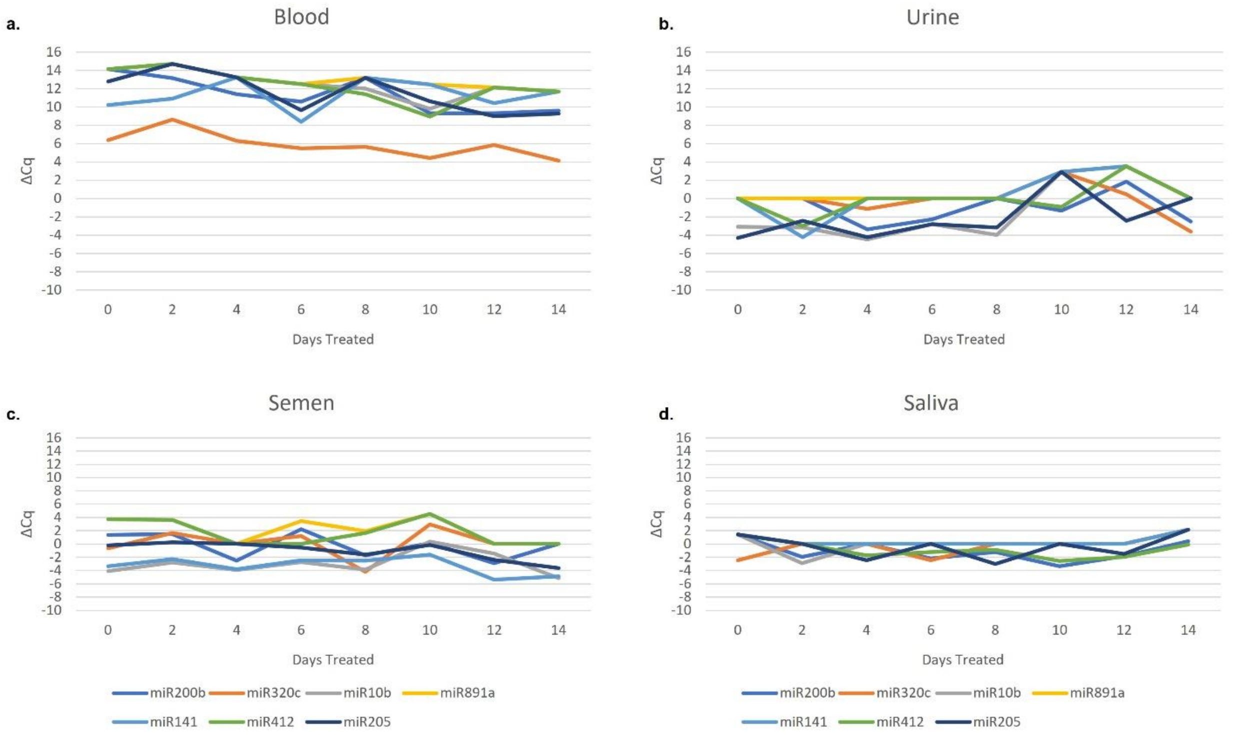

Supp Fig S2. Average dCq values of a. blood, b. urine, c. semen, and d. saliva, that were exposed to 0-14 days in a Q-sun Ce-3 Environmental Chamber (*n*=8 per body fluid).
