# Supplementary material for: Evaluation and Verification of a microRNA Panel Using Quadratic Discriminant Analysis for the Classification of Human Body Fluids in DNA Extracts
Source: Genes (Basel). 2023 Apr 25;14(5):968. doi: 10.3390/genes14050968 (PMC10218048; doi:10.3390/genes14050968)
Supplement: Supplementary file 1 [file genes-14-00968-s001.zip › Supp Table S1 - Env Chamber Temps.pdf]

Supplemental Table S1. Environmental Chamber Parameters on the Q-sun Ce-3 Environmental Chamber.

Step	Function	Irradiance (W/m^2)	BP Temp* (℃)	Air Temp (℃)	Humidity (%)	Time (h)
1	Light	0.34	45	35	50	3
2	Light	0.68	52	35	50	4
3	Light	0.34	45	35	50	3
4	Dark	–	–	32	50	14
5	Final Step, or Go to Step 1 (repeat for up to 14 days)					

*black panel temperature (BP Temp) is the Temperature of a sensor on the same level as the samples.
