# Supplementary material for: Evaluation and Verification of a microRNA Panel Using Quadratic Discriminant Analysis for the Classification of Human Body Fluids in DNA Extracts
Source: Genes (Basel). 2023 Apr 25;14(5):968. doi: 10.3390/genes14050968 (PMC10218048; doi:10.3390/genes14050968)
Supplement: Supplementary file 1 [file genes-14-00968-s001.zip › Supp Table S2 - miRNA primer table.pdf]

Supp Table S2. miRNA RT-qPCR targets

miRNA	miRbase accession ID	Mature miRNA sequence (5'-3')	qPCR forward primer sequence (5'-3')
hsa-let-7g-5p	MIMAT0000414	UGAGGUAGUAGUUUGUACAGUU	CCGAGCTGAGGTAGTAGTTTGTAC
hsa-let-7i-5p	MIMAT0000415	UGAGGUAGUAGUUUGUGCUGUU	CGTTCTGAGGTAGTAGTTTGTGCT
miR-200b-3p	MIMAT0000318	UAAUACUGCCUGGUAAUGAUGA	ACTGCCTGGTAATGATGAAAAA
miR-320c	MIMAT0005793	AAAAGCUGGGUUGAGAGGGU	AAAGCTGGGTTGAGAGGGT
miR-10b-5p	MIMAT0000254	UACCCUGUAGAACCGAAUUUGUG	CGTACCCTGTAGAACCGAATTTGT
miR-891a-5p	MIMAT0004902	UGCAACGAACCUGAGCCACUGA	CGAACCTGAGCCACTGAAA
miR-141-3p	MIMAT0000432	UAACACUGUCUGGUAAAGAUGG	CGTAACACTGTCTGGTAAAGATGGA
miR-412-3p	MIMAT0002170	ACUUCACCUGGUCCACUAGCCGU	CCTGGTCCACTAGCCGTAAA
miR-205-5p	MIMAT0000266	UCCUUCAUUCCACCGGAGUCUG	TCCTTCATTCCACCGGAGTC
