# Supplementary material for: Evaluation and Verification of a microRNA Panel Using Quadratic Discriminant Analysis for the Classification of Human Body Fluids in DNA Extracts
Source: Genes (Basel). 2023 Apr 25;14(5):968. doi: 10.3390/genes14050968 (PMC10218048; doi:10.3390/genes14050968)
Supplement: Supplementary file 1 [file genes-14-00968-s001.zip › Supp Table S3 - demographics.pdf]

Supp Table S3—Sample data including self-reported demographic information of individuals included in the expanded population study (Mens.=menstrual secretions, Vag.=vaginal secretions). N represents all individuals sampled for the project.

	**Blood**	**Mens.**	**Feces**	**Urine**	**Saliva**	**Semen**	**Vag.**
Individual donors	51	53	50	46	53	52	50
**Sex**							
Female	34	53	37	35	35	0	50
Male	17	0	13	11	18	52	0
**Age group (years)**							
< 18	1	0	0	1	0	0	0
18-30	44	51	48	42	46	42	46
31-50	5	0	1	1	6	5	3
> 50	0	0	0	0	1	2	1
Unreported	1	2	1	2	0	3	0
**Ethnicity**							
Caucasian	21	17	18	19	17	22	20
African American	14	16	18	12	14	14	11
Hispanic	4	7	4	5	4	2	4
Asian	5	8	8	6	8	8	10
Admixed	7	2	2	2	7	4	3
Other	0	2	0	2	3	1	2
Unreported	0	1	0	0	0	1	0
