# Supplementary material for: Evaluation and Verification of a microRNA Panel Using Quadratic Discriminant Analysis for the Classification of Human Body Fluids in DNA Extracts
Source: Genes (Basel). 2023 Apr 25;14(5):968. doi: 10.3390/genes14050968 (PMC10218048; doi:10.3390/genes14050968)
Supplement: Supplementary file 1 [file genes-14-00968-s001.zip › Supp Table S4 - confusion matrix v1.pdf]

Supp Table S4. Confusion matrix for QDA using 10-fold cross validation. The dataset consists of 845 observations (ΔCq values) including 500 simulated “Other” data points across four miRNA targets (miR-200b, miR-10b, miR-320c, miR-891a) that resulted in 88% overall correct classification rate. Bolded numbers indicate correct classifications (Mens.=menstrual secretions and Vag.=vaginal secretions).

		Predicted							
		Blood	Mens.	Feces	Urine	Saliva	Semen	Vag.	Other
**Actual**	Blood	**48**	0	0	0	1	0	0	0
	Mens.	0	**31**	0	0	8	0	10	1
	Feces	0	0	**44**	0	4	0	2	0
	Urine	0	0	0	**35**	2	6	3	0
	Saliva	0	1	5	4	**33**	0	7	0
	Semen	0	0	0	5	2	**43**	0	0
	Vag.	0	8	0	4	5	0	**33**	0
	Other	1	2	1	5	3	0	11	**477**
